# Manganese-Based Contrast Agents as Alternatives to Gadolinium: A Comprehensive Review

**DOI:** 10.3390/clinpract15080137

**Published:** 2025-07-25

**Authors:** Linda Poggiarelli, Caterina Bernetti, Luca Pugliese, Federico Greco, Bruno Beomonte Zobel, Carlo A. Mallio

**Affiliations:** 1 School of Medicine, Università Campus Bio-Medico di Roma, via Alvaro Del Portillo, 21, 00128 Rome, Italy; linda.poggia@gmail.com (L.P.); c.bernetti@policlinicocampus.it (C.B.); b.zobel@policlinicocampus.it (B.B.Z.); 2 Research Unit of Diagnostic Imaging, Department of Medicine and Surgery, Università Campus Bio-Medico di Roma, 00128 Rome, Italy; federico.greco@unicampus.it; 3 Radiology Unit, Department of Medical-Surgical Sciences and Translational Medicine, Sant’ Andrea University Hospital, Sapienza University of Rome, 00189 Rome, Italy; lpugliese@ospedalesantandrea.it; 4 Department of Radiology, Cittadella della Salute, Azienda Sanitaria Locale di Lecce, 73100 Lecce, Italy

**Keywords:** manganese, contrast agents, MRI, relaxivity

## Abstract

Background/Objectives: Magnetic resonance imaging (MRI) is a powerful, non-invasive diagnostic tool capable of capturing detailed anatomical and physiological information. MRI contrast agents enhance image contrast but, especially linear gadolinium-based compounds, have been associated with safety concerns. This has prompted interest in alternative contrast agents. Manganese-based contrast agents offer a promising substitute, owing to manganese’s favorable magnetic properties, natural biological role, and strong T1 relaxivity. This review aims to critically assess the structure, mechanisms, applications, and challenges of manganese-based contrast agents in MRI. Methods: This review synthesizes findings from preclinical and clinical studies involving various types of manganese-based contrast agents, including small-molecule chelates, nanoparticles, theranostic platforms, responsive agents, and controlled-release systems. Special attention is given to pharmacokinetics, biodistribution, and safety evaluations. Results: Mn-based agents demonstrate promising imaging capabilities, with some achieving relaxivity values comparable to gadolinium compounds. Targeted uptake mechanisms, such as hepatocyte-specific transport via organic anion-transporting polypeptides, allow for enhanced tissue contrast. However, concerns remain regarding the in vivo release of free Mn^2+^ ions, which could lead to toxicity. Preliminary toxicity assessments report low cytotoxicity, but further comprehensive long-term safety studies should be carried out. Conclusions: Manganese-based contrast agents present a potential alternative to gadolinium-based MRI agents pending further validation. Despite promising imaging performance and biocompatibility, further investigation into stability and safety is essential. Additional research is needed to facilitate the clinical translation of these agents.

## 1. Introduction to MRI and Contrast Agents

Magnetic resonance imaging (MRI) is a key non-invasive imaging technique used in both clinical practice and biomedical research [1]. The importance is partly due to its capability to generate high-resolution, three-dimensional images of tissues [1]. Remarkably, MRI uniquely captures both anatomical detail and a wide range of physiological parameters in a single session—capabilities unmatched by other imaging modalities [2].

MRI is based on the slight difference in the population of protons aligned with and against a strong magnetic field, resulting in a small net magnetization under thermal equilibrium conditions. MRI detects radiofrequency (RF) signals from the magnetic moments of hydrogen protons, mainly in water and lipids within biological tissues [3,4,5,6]. Water serves as an ideal molecular probe for several reasons. Hydrogen nuclei have the highest sensitivity among biologically relevant atoms, and water is abundantly present in nearly all tissues. Moreover, water’s magnetic behavior is influenced by the microstructural and compositional characteristics of tissues [2].

Dynamic biological processes such as diffusion [7,8], perfusion [9], blood flow [10,11], and tissue motion [12] can be captured using specialized MRI sequences. Furthermore, the magnetic environment around hydrogen nuclei in water can be altered by magnetic agents, whether naturally occurring (such as deoxyhemoglobin in blood) or externally administered in the form of contrast agents [13]. These factors contribute to MRI’s wide range of contrast mechanisms, which are a hallmark of the modality [2].

In conventional MR imaging, tissue contrast primarily arises from proton density and the longitudinal (T1) and transverse (T2 and T2*) relaxation times. In many cases, healthy soft tissues exhibit sufficient variation in T1 and T2 relaxation characteristics to allow clear anatomical differentiation in T1- or T2-weighted scans. Pathological tissues can also be identified by their distinct relaxation properties. However, certain diseases may not produce obvious morphological differences or notable alterations in relaxation times. In such cases, the use of MRI contrast agents becomes valuable, as they locally influence relaxation behavior to highlight affected tissues and can provide substantial diagnostic benefits [2].

Gd-based contrast agents can influence the T1 relaxation time (which are usually paramagnetic metal ion-containing agents) and T2 relaxation time (known as superparamagnetic contrast agents) contrast agents [14].

Manganese-based contrast agents have emerged as a promising alternative to gadolinium-based contrast agents [15], offering potential advantages due to manganese’s natural biological role and favorable magnetic properties [16]. Gadolinium-based contrast agents account for the vast majority of clinical MRI contrast use, with millions of doses administered annually worldwide [17]. However, concerns persist with linear gadolinium-based contrast agents regarding their potential for nephrogenic systemic fibrosis (NSF) and gadolinium deposition in the brain, particularly within the dentate nucleus and globus pallidus, likely due to transmetallation or incomplete clearance of dissociated Gd^3+^ ions [1,13,18,19].

Manganese-based contrast agents exhibit strong T1 relaxivity, biocompatibility, and potential for targeted imaging applications, making them highly attractive for both clinical and research settings [16].

Given the growing interest in manganese chemistry, the development of novel manganese-based contrast agents, and their expanding range of biomedical applications, a comprehensive review of this field is timely. In this review, we examine the design, mechanisms, applications, and current challenges associated with manganese-based contrast agents, providing a critical overview of their potential to shape the future of diagnostic imaging.

## 2. Manganese-Based MRI Contrast Agents

MRI frequently relies on contrast agents to improve the visibility of pathological tissues. Among the most commonly used agents in clinical practice are gadolinium chelates, which enhance the longitudinal relaxation of water protons [17]. However, it has long been established that administering linear Gd-based agents to patients with impaired renal function can trigger a condition known as NSF [18,19,20,21]. Although this issue has been largely addressed by more stable gadolinium-based macrocyclic compounds, it has prompted the search for alternatives that do not rely on gadolinium, such as magnetic nanoparticles [22], fluorine-based MRI techniques [23], nitroxide radical systems devoid of metals [24], and manganese-based contrast agents [25,26,27,28].

Manganese-based contrast agents can be classified into the following five main categories:

1. Small-molecule agents, such as low-molecular-weight chelates of Mn^2+^, designed to mimic gadolinium agents, are suitable for dynamic imaging but may pose stability concerns. Examples: Mn-DPDP and Mn-PyC3A [29,30].
2. Manganese-based nanoparticles, i.e., nanostructures incorporating Mn for high relaxivity and multifunctionality, are often used for passive or active tumor targeting. Examples: MnO nanoparticles and Mn-doped silica NPs [31,32].
3. Theranostic agents are used for diagnostic imaging with therapeutic capabilities, such as drug delivery or photothermal therapy. Examples: MnO@SiO_2_ NPs loaded with drugs or photosensitizers [32,33].
4. Responsive (activatable) agents, such as Mn complexes that release Mn^2+^ or change relaxivity in response to specific stimuli (e.g., pH, enzymes, and redox environment). Examples: Mn-Tyr-EDTA and redox-responsive Mn chelates [32].
5. Controlled-release systems, or rather carriers engineered to gradually release Mn^2+^ at target sites, aiming to prolong contrast effect and reduce systemic toxicity. Examples: polymeric or liposomal Mn formulations [31].

In biological systems, manganese typically exists as Mn^2+^ or Mn^3+^, with five or four unpaired d-electrons, respectively. Manganese plays key roles as a cofactor in enzymatic activation, contributes to metalloenzyme structure, and is involved in immune and nervous system development as well as the regulation of glucose and vitamin levels in the blood [34,35,36,37,38].

**Figure 1:**
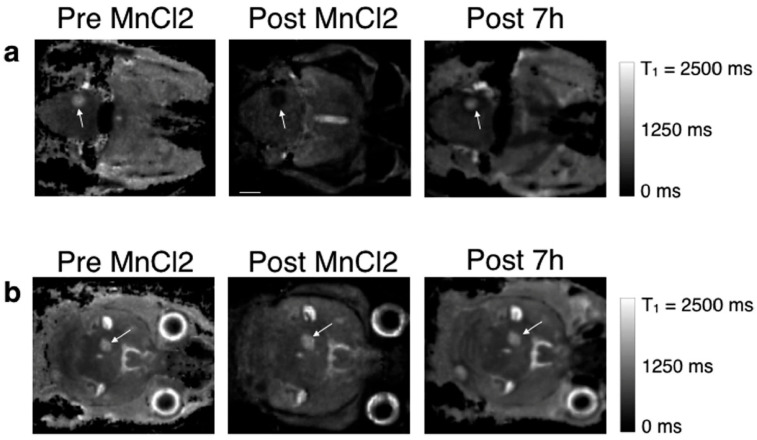
Three-dimensional T1 maps of a mouse brain displaying metastases at multiple time points after MnCl_2_ administration. The images show two slices, each featuring a metastasis of similar size (marked by arrows). In (**a**), the metastasis demonstrates Mn^2+^ uptake, whereas in (**b**), the T1 values remain consistent over time. The scale bar indicates 2 mm. Reproduced from reference [36] under the Creative Commons Attribution 4.0 International License.

**Figure 2:**
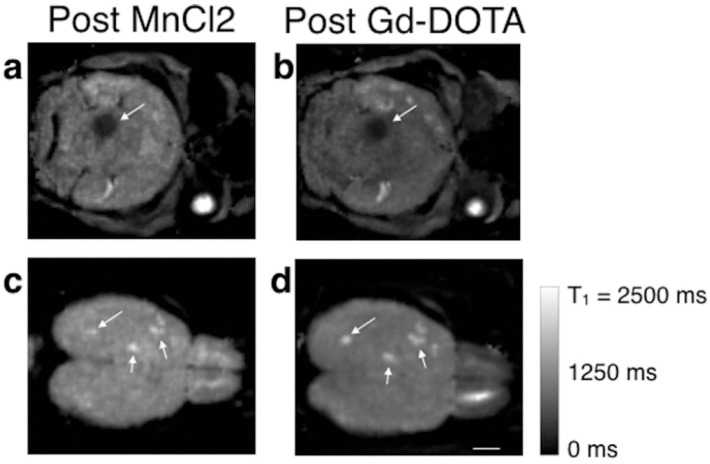
Three-dimensional T1 maps of mouse brains with metastases acquired immediately following MnCl_2_ or Gd-DOTA injection. Metastases that exhibited Mn^2+^ uptake (arrow in (**a**)) corresponded to regions with a leaky vasculature, as indicated by reduced T1 values after Gd-DOTA administration (**b**). In contrast, metastases with an intact blood–tumor barrier (BTB) did not take up either Mn^2+^ or Gd-DOTA (arrows in (**c**,**d**)). The scale bar represents 2 mm. Reproduced from reference [36] under the Creative Commons Attribution 4.0 International License.

The first manganese-based MRI contrast agent introduced was an oral formulation containing MnCl_2_ (LumenHance^®^), used for gastrointestinal imaging. However, excessive doses of free manganese were found to cause manganism, a neurotoxic condition resembling Parkinson’s disease, leading to its withdrawal from clinical use. Despite this, manganese-enhanced MRI (MEMRI) with MnCl_2_ remains widely used in preclinical studies, especially in mouse models of brain [36] and lung tumors [28,37] (Figure 1 and Figure 2).

To ensure safer clinical applications, manganese chelates have been synthesized. One such example is manganese dipyridoxyl diphosphate (Mn-DPDP, Teslascan^®^), which became the second Mn-based contrast agent to gain FDA approval in 1997 for liver-specific imaging [38,39]. However, this compound demonstrated concerns mainly over free Mn ion release in vivo, resulting in its removal from the market. Consequently, there remains an ongoing demand for contrast agents that combine strong thermodynamic stability, kinetic robustness, and effective imaging performance [25,28,40,41,42].

Similar to gadolinium-based complexes, the functionality of molecular Mn-based contrast agents depends on the presence of at least one inner-sphere water molecule that exchanges rapidly with the bulk solvent. Mn^2+^ typically adopts coordination numbers of six to eight in aqueous solutions, and for an inner-sphere water molecule to be maintained, the coordinating ligand must occupy five or six coordination sites. Nonetheless, Mn complexes often exhibit lower thermodynamic and kinetic stability compared to Gd-based compounds due to the lower charge of Mn ions and the absence of ligand-field stabilization associated with their high-spin d^5^ configuration. Furthermore, oxidation of Mn^2+^ to Mn^3+^ must be avoided, as this reduces the number of unpaired electrons, thereby diminishing relaxivity and contrast efficiency [28].

Several strategies have been developed to improve the kinetic and thermodynamic stability of Mn-based agents, thereby mitigating the risk of free Mn^2+^ ion release. Macrocyclic ligands such as NOTA and PyC3A exhibit high chelation strength and inertness, making them favorable candidates for clinical translation [29,30]. Additionally, chelators with planar tetradentate scaffolds, such as those derived from 1,2-phenylenediamido ligands, stabilize Mn in a configuration that retains strong relaxivity while preventing oxidation-induced loss of efficacy [43]. This framework somehow parallels the differences between linear and macrocyclic Gd-based agents, driven by similar safety concerns [44]. The increased stability of macrocyclic structures makes them promising candidates for safer clinical use of Mn-based MRI contrast agents [28].

These approaches collectively aim to enhance the safety profile of Mn-based agents by reducing the likelihood of Mn^2+^ dissociation in vivo [28].

Despite these challenges, some Mn^3+^-based complexes have been reported [43], particularly those involving planar tetradentate ligands derived from a 1,2-phenylenediamido scaffold.

To achieve a balance between stability and efficacy, numerous Mn-coordinating ligands have been developed, including both linear and macrocyclic designs [25,40,41]. These Mn complexes are generally non-targeted and quickly eliminated via renal pathways, though some are engineered to localize in specific tissues such as tumors or the liver. Additionally, certain formulations have been designed to be stimulus-responsive or to support multimodal imaging approaches [28].

### Manganese Pharmacokinetics and Toxicity

To date, only a limited number of manganese complexes have undergone preclinical evaluation (Table 1). Interestingly, many of these complexes exhibit increased lipophilicity, which facilitates their application as liver-specific or blood pool MRI contrast agents. MRI- and ICP-based biodistribution analyses reveal that these agents are eliminated via both renal and hepatobiliary pathways, a pattern attributed to their higher lipophilicity compared to small extracellular agents such as Gd-DOTA. Notably, Mn-PyC3A was tested in a rat model with renal dysfunction, where results indicated enhanced hepatobiliary clearance [29]. Furthermore, Mn concentrations in all tested agents returned to baseline within 24 h post-injection [28].

These complexes have also been evaluated for their effectiveness as MRI contrast agents. In particular, liver-targeting compounds were tested in a mouse liver tumor model to determine their ability to distinguish between healthy and cancerous liver tissue. Post-injection MRI scans generally revealed a hypointense signal in tumor areas relative to normal liver, which is likely due to the uptake mechanism of these agents: Mn complexes are taken up by healthy hepatocytes through organic anion-transporting polypeptides (OATPs), which are significantly downregulated in tumor tissue. Chen et al. [33] emphasized the role of OATPs in hepatic uptake by using OATP inhibitors and performing uptake studies on cell lines with and without OATP expression. However, findings on Mn-NOTA-NP [30] contradicted this trend, as a hyperintense signal was observed in tumors compared to normal liver. The authors attributed this result to reduced expression of MRP2 in tumor cells, while OATP levels remained stable. Since MRP2 facilitates the export of Mn complexes from cells, its downregulation may cause intracellular accumulation of Mn complexes, resulting in a hyperintense signal, and thereby highlighting the complexity of these mechanisms and the need for further investigation [28].

The involvement of OATP and MRP2 transporters is critical for understanding Mn-based agent biodistribution and contrast behavior. OATP-mediated hepatic uptake and MRP2-linked efflux influence signal intensity in tumors, depending on their expression levels. These mechanisms have diagnostic implications but may also limit clinical translation due to inter-patient and inter-tumoral variability [28,30,33].

Some Mn-based blood pool agents have also been studied in vivo for their imaging capabilities. For instance, Mn-LCyPh2 was administered to rabbits at doses of 10 and 30 µmol/kg, yielding clear vascular imaging at both levels and allowing differentiation between healthy and damaged vessels [31]. In another study, Mn-DO2AM-Gly showed promising contrast enhancement in a highly vascularized breast tumor model [32]. Additionally, Mn-Tyr-EDTA was tested in a mouse model of acute gouty arthritis, showing higher signal intensity at inflammation sites than that achieved with Gd-DTPA [28].

Despite these promising findings, data on the toxicity of Mn-based agents remain scarce. A few studies have included cell viability assays [30,32,33,46] to assess cytotoxicity, generally reporting minimal toxicity across different cell lines at clinically relevant concentrations. However, such preliminary data are not sufficient to establish in vivo safety. As with gadolinium-based agents, the potential release of free Mn^2+^ ions could pose serious health risks, particularly the risk of manganism, a condition with neurological symptoms resembling Parkinson’s disease.

Physiological serum concentrations of manganese in healthy individuals range from approximately 0.5 to 1.2 μg/dL (9–22 μM) [28,34]. Neurotoxicity typically occurs when manganese accumulates in the brain beyond these levels, especially with chronic or high-dose exposure. Preclinical studies have shown that several manganese-based contrast agents return to baseline Mn concentrations within 24 h post-injection and exhibit low cytotoxicity at imaging-relevant doses [28,29]. However, this rapid clearance and low acute cytotoxicity do not negate the importance of ensuring the manganese remains stably chelated, as to minimize the risk of toxicity, it is critical to ensure that Mn^2+^ remains tightly bound within stable complexes and that administered doses remain well below thresholds associated with adverse neurological effects.

In a study [31], the authors suggested that Mn-LCyPh2 remained intact, reporting the absence of acute cardiotoxicity, a notable observation given the known cardiac toxicity of free Mn^2+^ ions. Still, this constitutes indirect evidence, and more comprehensive safety studies, similar to those conducted for gadolinium agents following concerns over NSF and brain retention, are necessary to fully evaluate the safety profile of Mn-based contrast agents [28].

In addition to preclinical studies, some manganese-based contrast agents have entered clinical evaluation. Mn-PyC3A, for example, has progressed to Phase I/II human trials, where its safety, biodistribution, and clearance were assessed using PET-MRI in healthy subjects and patients [28,29]. These early clinical results are promising and represent an important step toward the clinical adoption of stabilized Mn-based contrast agents (Table 2).

## 3. Future Perspectives

Many of the previously mentioned examples of Mn complexes involve the release of free Mn^2+^ ions, which may raise toxicity concerns that have not yet been thoroughly investigated. While in vivo studies generally report no signs of toxicity from Mn-based nanoparticles at specific concentrations, indicating low cytotoxicity and favorable biocompatibility, there is evidence suggesting that higher doses could increase their toxic potential. Additionally, the long-term impact of Mn nanoparticle exposure remains largely unexplored. Once released, Mn^2+^ ions may interact with the body’s natural manganese pathways, potentially leading to delayed or hidden effects. Therefore, before any of these Mn-based systems can be considered for clinical use, comprehensive safety assessments will be essential [28].

Extensive research efforts have been devoted to the development of manganese-based MRI contrast agents as a potentially safer alternative to the currently used gadolinium compounds. Both small-molecule and nanoparticle-based agents have been explored. In the case of molecular agents, some have demonstrated relaxivity values comparable to or even exceeding those of gadolinium-based agents. However, ensuring adequate thermodynamic stability and kinetic inertness remains a critical consideration. Several of the discussed studies stand out in this regard and may represent promising candidates for eventual clinical application [28].

As for nanoparticle systems, their development is equally compelling due to their exceptionally high relaxivity and the relative ease with which they can be functionalized with targeting or therapeutic groups. Nevertheless, thorough toxicity evaluations will be necessary before these systems can be safely translated to clinical practice. This requirement also applies to theranostic platforms, which hold considerable potential for the simultaneous imaging and treatment of tumors.

A major safety concern associated with manganese-based contrast agents is the potential release of free Mn^2+^ ions, which may lead to neurotoxicity, particularly a condition known as manganism—a Parkinson-like syndrome resulting from excessive manganese accumulation in the brain [28,34,46,47]. Although several studies have demonstrated rapid clearance of Mn-based agents and minimal cytotoxicity at clinically relevant doses [28,29,46,47], these findings are largely limited to acute toxicity. Long-term safety data remain scarce, and the effects of repeated or high-dose exposure have not been thoroughly evaluated. Therefore, ensuring that Mn^2+^ remains tightly chelated and that dosing remains within safe limits is critical to mitigating neurological risks.

With additional research and rigorous preclinical evaluation, certain manganese-based agents—particularly those demonstrating high relaxivity, favorable biodistribution, and low toxicity—may hold promise for future clinical application. However, their successful translation will depend on comprehensive safety assessments and regulatory validation.

Achieving clinical approval for manganese-based MRI contrast agents presents significant hurdles. Key challenges include demonstrating comprehensive long-term safety, particularly regarding neurotoxicity and other organ toxicities. Crucial is ensuring robust thermodynamic and kinetic stability to prevent in vivo Mn^2+^ release.

For regulatory approval (EMA/FDA), extensive toxicology and pharmacokinetic data are mandatory. Well-powered clinical trials must show diagnostic efficacy, ideally through direct comparison to approved gadolinium agents. Endpoints typically cover sensitivity, specificity, and long-term safety, all conducted under strict Good Laboratory and Clinical Practice (GLP/GCP) standards.

Despite the expanding body of literature describing the pharmacological, imaging, and safety profiles of manganese-based MRI contrast agents, a notable gap remains in the evaluation of their cost-effectiveness. Indeed, there is a lack of knowledge on production costs, clinical implementation costs, or potential economic benefits associated with the usage of manganese-based MRI contrast agents. Comprehensive health-economic analyses, including cost–utility and cost–benefit modeling, will be essential to inform the broader adoption of manganese-based agents in clinical imaging workflows.

## 4. Conclusions

Manganese-based contrast agents may offer a promising alternative to gadolinium-based agents in MRI, with high T1 relaxivity and the potential for targeted imaging. Diverse structures, including small molecules and nanoparticles, show promise for enhanced diagnostic specificity in various pathologies. However, their successful clinical translation is contingent upon comprehensive safety assessments, particularly concerning the stability of manganese complexes and the potential for long-term toxicity. Future research must prioritize rigorous preclinical evaluation to ensure their safe and effective integration into diagnostic imaging.

## Figures and Tables

**Table 1:** Toxicity of manganese-based contrast agents in relevant preclinical studies. Abbreviations: magnetic resonance imaging (MRI), manganese (II) ion (Mn^2+^), organic anion-transporting polypeptide (OATP), multidrug resistance-associated protein 2 (MRP2), nanoparticle (NP), gadolinium (Gd), positron emission tomography (PET), longitudinal relaxation time (T1), transverse relaxation time (T2), micromoles per kilogram (μmol/kg), millimoles per kilogram (mmol/kg), in the living organism (in vivo), outside the living organism (in vitro), intravenous (IV), hour (h), dipyridoxyl diphosphate (DPDP), and pyridine-carboxylate-triacetate (PyC3A).

Investigators and Year	Country	In Vitro/In Vivo	Cohorts or Model	Study Design	Dose/Follow-Up	Toxicity/Model Details	Main Findings	Conclusions
Chen et al., 2021 [33]	China	In vitro and in vivo	OATP-transfected cell lines; mouse liver tumor model	Mechanistic imaging and transport study	0.1 mM Mn agent; 24 h follow-up	Hepatic uptake assessed; mouse (female, −20–25 g); no acute neurotoxicity reported	Demonstrated OATP-mediated uptake of Mn complexes in normal liver; reduced uptake in tumors using inhibitor	OATPs are key in liver-specific targeting of Mn agents
Islam et al., 2023 [30]	Korea	In vivo	Mouse liver tumor model	Comparative tumor imaging study	0.05 mmol/kg; imaging up to 1 h post-injection	MRP2-linked retention; mouse model (−25 g), no explicit toxicity measured	Observed hyperintense signal in tumors; MRP2 downregulation caused Mn accumulation	Tumor MRP2 levels influence Mn complex retention; uptake mechanisms may vary across agents
Zhou et al., 2021 [29]	USA	In vivo	Rat model with renal dysfunction	Biodistribution in a renal impairment model	0.2 mmol/kg; 1- and 7-day PET-MRI follow-ups	Renal vs. hepatic excretion; adult rats (−250 g); systemic clearance tracked	Increased hepatobiliary clearance of Mn-PyC3A in impaired renal function	Hepatic clearance may compensate in renal impairment
Troughton et al., 2004 [31]	USA	In vivo	New Zealand white rabbits	Blood pool imaging and safety-focused imaging study	10 and 30 µmol/kg; acute evaluation after administration	Rabbit (−3 kg); no acute cardiotoxicity; protein binding and vascular imaging	Effective vascular imaging at 10 and 30 µmol/kg; no acute cardiac toxicity observed	Suggests complex stability; more direct safety data required
Leone et al., 2022 [32]	Italy	In vivo	Mouse breast tumor model	Tumor vascular imaging	0.1 mmol/kg; imaging within 1 h post-injection	Mouse (−25 g); no systemic toxicity reported	Strong contrast enhancement in vascularized tumor tissue	Potential agent for tumor imaging
Islam et al., 2017 [45]	Korea	In vitro and in vivo	Mouse model (for in vivo imaging studies)	Relaxivity measurements in vitro and MRI and biodistribution analysis in vivo	0.05 mmol/kg; follow-up up to 24 h	Liver contrast and biodistribution in mice; low cytotoxicity in vitro	High liver uptake and strong T1 MRI contrast enhancement	Promising liver-specific MRI contrast agent alternative to Gd-based agents

**Table 2:** Summary of manganese-based MRI contrast agent classes, detailing their structural characteristics, targeting strategies, relaxivity values, toxicity considerations, and translational status. Abbreviations: magnetic resonance imaging (MRI), manganese (Mn), gadolinium (Gd), organic anion-transporting polypeptides (OATPs), human serum albumin (HSA), enhanced permeability and retention (EPR), longitudinal relaxivity (r_1_), nanoparticle (NP), Food and Drug Administration (FDA), European Medicines Agency (EMA), tetraazacyclododecane-1,4-diacetic acid (DO2A), N,N-dimethylacetamide derivative of DO2A (DO2AM), 1,4,7-triazacyclononane-1,4,7-triacetic acid (NOTA), dipyridoxyl diphosphate (DPDP), and longitudinal relaxation time (T1).

Mn-Agent Class	Structural Type	Targeting Strategy	Relaxivity (r1, mM^−1^s^−1^)	Toxicity Status	Translational Stage
Small Molecule (e.g., Mn-DPDP and Mn-EDTA-BTA)	Linear chelates	Passive liver uptake via OATPs	1.5–3.0	Generally low; concerns with free Mn^2+^ release	One agent (Mn-DPDP) was FDA/EMA-approved but is now withdrawn
Macrocyclic Chelates (e.g., Mn-NOTA-NP and Mn-DO2AM-Bn)	Macrocyclic ligands with hydrophobic moieties	HSA binding and hepatocyte targeting	3.5–9.0 (depending on protein binding)	Good in vitro/in vivo profile; enhanced stability	Preclinical and early clinical trials (e.g., Mn-PyC3A)
Nanoparticles (e.g., MnO NPs)	Inorganic or hybrid nanoformulations	Passive or active targeting (e.g., EPR effect)	Up to 30 (per particle); variable	Low acute toxicity; limited long-term data	Mostly preclinical
Theranostic Platforms (e.g., MnO@SiO_2_-drug)	Nanoparticle-based carriers with dual function	Targeted delivery and therapy (e.g., tumor markers)	Variable; often enhanced due to NP matrix	Preclinical safety under evaluation	Preclinical proof-of-concept
Responsive Agents (e.g., Mn-Tyr-EDTA)	Stimulus-sensitive molecular constructs	Environment-triggered activation (pH and redox)	Moderate; stimulus-dependent	Preclinical studies; limited systemic data	Preclinical models
Controlled-Release Systems (e.g., Mn-liposomes)	Encapsulated Mn^2+^ in biodegradable carriers	Slow Mn^2+^ release at target tissue	Sustained enhancement; moderate	Minimized systemic toxicity by design	Early stage, preclinical

## Abbreviations

MRI	magnetic resonance imaging
RF	radiofrequency
CEST	Chemical Exchange Saturation Transfer
PET	Positron Emission Tomography
SPECT	Single-Photon Emission Computed Tomography
NSF	nephrogenic systemic fibrosis
MEMRI	manganese-enhanced MRI
OATPs	organic anion-transporting polypeptides
